# Low Innate Immunity and Lagged Adaptive Immune Response in the Re-Tested Viral RNA Positivity of a COVID-19 Patient

**DOI:** 10.3389/fimmu.2021.664619

**Published:** 2021-07-01

**Authors:** Changchun Lai, Xinglong Liu, Qihong Yan, Hualiang Lv, Lei Zhou, Longbo Hu, Yong Cai, Guoqiang Wang, Yufeng Chen, Renjie Chai, Zhenwei Liu, Yuhua Xu, Wendong Huang, Fei Xiao, Linhui Hu, Yaocai Li, Jianhong Huang, Qiang Zhou, Luqian Li, Tao Peng, Haiye Zhang, Zhenhui Zhang, Ling Chen, Chunbo Chen, Tianxing Ji

**Affiliations:** ^1^ Clinical Laboratory Medicine Department, Maoming People’s Hospital, Maoming, China; ^2^ Department of Emergency, Maoming People’s Hospital, Maoming, China; ^3^ Clinical Laboratory Medicine Department, Xinyi People’s Hospital, Xinyi, China; ^4^ Guangzhou Regenerative Medicine and Health-Guangdong Laboratory (GRMH-GDL), Guangzhou Institutes of Biomedicine and Health, Chinese Academy of Sciences, Guangzhou, China; ^5^ Pulmonary and Critical Care Medicine Department, Maoming People’s Hospital, Maoming, China; ^6^ Pathology Laboratory Department, Maoming People’s Hospital, Maoming, China; ^7^ State Key Laboratory of Respiratory Disease, Sino-French Hoffmann Institute, School of Basic Medical Science, Guangzhou Medical University, Guangzhou, China; ^8^ CT Department, Maoming People’s Hospital, Maoming, China; ^9^ Department of Gastrointestinal Surgery, The Second Affiliated Hospital of Guangzhou Medical University, Guangzhou, China; ^10^ Cardiovascular Department, The Second Affiliated Hospital of Guangzhou Medical University, Guangzhou, China; ^11^ State Key Laboratory of Respiratory Disease, National Clinical Research Center for Respiratory Disease, Guangzhou Institute of Respiratory Health, The First Affiliated Hospital of Guangzhou Medical University, Guangzhou, China; ^12^ Vaccine Research and Development Department, Guangdong South China Vaccine Co. Ltd, Guangzhou, China; ^13^ Scientific Research Center, Maoming People’s Hospital, Maoming, China; ^14^ Clinical Research Center, Maoming People’s Hospital, Maoming, China; ^15^ Infection Department, Maoming People’s Hospital, Maoming, China; ^16^ Medical Department, Maoming People’s Hospital, Maoming, China; ^17^ Clinical Laboratory Medicine Department, The Second Affiliated Hospital of Guangzhou Medical University, Guangzhou, China; ^18^ Critical Care Medicine Department, The Second Affiliated Hospital of Guangzhou Medical University, Guangzhou, China; ^19^ Bioland Laboratory (GRMH-GDL), Guangzhou Institutes of Biomedicine and Health, Chinese Academy of Sciences, Guangzhou, China; ^20^ State Key Laboratory of Respiratory Disease, Guangzhou Institute of Respiratory Health, First Affiliated Hospital of Guangzhou Medical University, Guangzhou, China

**Keywords:** delayed neutralizing antibody response, delayed antigen presentation, delayed anti-RBD/anti-spike IgA conversion, SARS-COV-2, COVID-19, re-tested positivity

## Abstract

Recent studies have highlighted observations regarding re-tested positivity (RP) of SARS-CoV-2 RNA in discharged COVID-19 patients, however, the immune mechanisms underlying SARS-CoV-2 RNA RP in immunocompetent patients remain elusive. Herein, we describe the case of an immunocompetent COVID-19 patient with moderate symptoms who was twice re-tested as positive for SARS-CoV-2 RNA, and the period between first and third viral RNA positivity was 95 days, longer than previously reported (18–25 days). The chest computed tomography findings, plasma anti-SARS-CoV-2 antibody, neutralizing antibodies (NAbs) titer, and whole blood transcriptic characteristics in the viral RNA RP patient and other COVID-19 patients were analyzed. During the SARS-CoV-2 RNA RP period, new lung lesions were observed. The COVID-19 patient with viral RNA RP had delayed seroconversion of anti-spike/receptor-binding domain (RBD) IgA antibody and NAbs and were accompanied with disappearance of the lung lesions. Further experimental data validated that NAbs titer was significantly associated with anti-RBD IgA and IgG, and anti-spike IgG. The RP patient had lower interferon-, T cells- and B cell-related genes expression than non-RP patients with mild-to-moderate symptoms, and displayed lower cytokines and chemokines gene expression than severe patients. Interestingly, the RP patient had low expression of antigen presentation-related genes and low B cell counts which might have contributed to the delayed anti-RBD specific antibody and low CD8+ cell response. Collectively, delayed antigen presentation-related gene expression was found related to delayed adaptive immune response and contributed to the SARS-CoV-2 RNA RP in this described immunocompetent patient.

## Introduction

Recent reports have highlighted the recurrence of SARS-CoV-2 RNA positivity in discharged COVID-19 patients (1) and their risk of being infectious (2). The causes for recurrent positive detection of SARS-CoV-2 RNA might be low virus load in the respiratory samples of discharged COVID-19 patients and/or low assay-sensitivity of detection methods; leading to false negative results at the time of discharge (3). Therefore, the “turned positive” phenomenon of SARS-CoV-2 RNA could be indicating prolonged viral shedding rather than “recurrence” (4). More importantly, SARS-CoV-2 could accelerate its mutation during prolonged infection in immunocompromised COVID-19 patients, particularly, the mutation of receptor-binding domain (RBD) region of spike gene could lead to immunologic escape, then bring about novel higher infectious virus isolates emergence, leading to recurrence of infection or fatality (5). Moreover, infectious virus could be shedded in few SARS-CoV-2 RNA redetectable patients (6). Therefore, there is great urgency to promptly identify these patients to implement timely therapy and quarantine measures.

Prolonged SARS-CoV-2 RNA shedding has been mostly reported in immunocompromised patients (7–10). These studies demonstrated that a decline or delay in antibody response could lead to relapse or recovery of COVID-19 (7). However, in a recent report involving a lymphoma patient with low immunity response, the patients were re-tested as positive for SARS-CoV-2 RNA after administration of COVID-19 convalescent plasma transfusion (11). Additionally, prolonged SARS-CoV-2 RNA shedding was observed after seroconversion of anti-spike antibody in a COVID-19 patient (3), suggesting that other mechanism attributes, apart from antibody, could also be playing key roles in virus clearance (12). Multiple evidences suggest that cellular immunity also played critical roles in SARS-CoV-2 clearance (13); thereby, relating the reduction of CD8^+^ T-cell as a potential risk factor for the longer duration of SARS-CoV-2 RNA positivity (14). Herein, we explored the clinical characteristics of a COVID-19 patient who was twice re-tested as positive for SARS-CoV-2 RNA and the immune mechanisms underlying re-tested viral RNA positivity in this immunocompetent patients.

## Methods

### Patients and Samples

The study was conducted in accordance with the Declaration of Helsinki and was approved by the Ethics Committee of Maoming People’s Hospital (Approval No. 2020012). The patient’s clinical data were extracted from the hospital records. The severity of the disease was assessed using the Seventh Version of the Novel Coronavirus Pneumonia Diagnosis and Treatment Guidance from the National Health Commission of China (15). Plasma at viral RNA positive and negative stage of each patient was collected for antibodies detection. In addition, 1mL of whole blood was added to 4 ml trizol solution (Invitrogen, USA), then stored at −80°C for further transcriptome analysis.

### Purification of IgA and IgG From Anti-RBD Antibody-Positive Plasma

The IgA was isolated using Peptide M/Agarose (Invivogen, USA). Briefly, 1 ml of Peptide M/Agarose was packed into a suitable column, then equilibrated with 5 ml equilibration and wash buffer (EWB). Afterward, 0.9 ml of plasma was loaded into the column and was washed with 10 ml of EWB. The binding antibody IgA was eluted with 10 ml of elution buffer. Subsequently, neutralization buffer was added into the elution buffer containing IgA to adjust pH to 7.5. Then, the plasma with IgA was loaded onto the Protein A column (GE, 17508001) and chromatography were performed at a flow rate of 5 ml/min using the AKTA Z100 (GE). Next, the columns were washed with PBS. IgG was eluted with 10 ml of 0.1 M glycine (Scientific Chemical, VA13110) and the pH was immediately adjusted to 7.5 with 1 M of Tris (Sigma-Aldrich, T6606). Finally, PBS exchange was achieved using Amicon Ultra centrifugal filters (Merck Millipore, UFC9050) through a 50-kD membrane according to the manufacturer’s instructions. The absorbance value of anti-RBD/anti-spike IgM, IgG, and IgA were determined using the above ELISA method in a dilution of 1:200.

### Anti-NP, Anti-RBD, Anti-Spike Antibody Detection

Antibody detection was performed as previously described (16). Briefly, 100 μl of PBS containing 50 ng of NP, RBD or S1/S2 mixture (Sino Biological, Beijing, China) was added into the wells of 96-well polystyrene plates and were incubated at 4°C overnight. After two washes with PBST, 100 μl of phosphate-buffered saline with 0.1% tween^®^20 (PBST) containing 5% non-fat milk was added and incubated at 37°C; for 1 h. Diluted plasma (1:200) was then added into the wells after three washings with PBST and incubated at 37°C for 2 h. Some 100 μl of diluted Anti-Human IgG-HRP (1:10,000, Sigma Aldrich, USA), Anti-Human IgM-HRP (1:2,000, Sigma Aldrich, USA), or Goat Anti-Human IgA-HRP (1:40,000, SouthernBiotech, USA) was then added into the wells after four washings with PBST and incubated at 37°C for 1 h. The 96-well polystyrene plates were washed five times with PBST, then 50 μl of TMB (3, 3′, 5,5′-Tetramethylbenzidine) One Solution (Guangzhou Jingxin Biotechnology Co., Ltd, China) was added and incubated at room temperature for 5–15 min. Next, 50 μl of 1 M H_2_SO_4_ solution was added to terminate the chromogenic reaction. The ratios of absorbance were calculated by the absorbance value dividing 2.1 × mean absorbance value of four negative control pooled plasma samples (10 SARS-CoV-2 RNA negative plasma samples were pooled as one pool sample).

### Pseudotyped Virus-Based Neutralization Assay

Neutralization assay was performed according to our previously described methods (17). In brief, lentiviral vectors pseudotyped by an S protein were produced by co-transfecting three plasmids: a transfer plasmid, a packaging plasmid, and an envelope plasmid expressing SARS-CoV-2 S protein, into 293 T cells (ATCC^®^ CRL-3216™). The plasma and immunoglobulins were serially diluted and incubated with pseudotyped virus for 1 h at 37°C. Subsequently, the mixture was added to 293 T/ACE2 in 96-well plates for infection of cells. After 48 h, luciferase activity in the cell lysates was examined with a luciferase assay system (Vazyme, DD1201). The titers of neutralizing antibodies (Nabs) were calculated as 50% inhibitory concentration (IC50), expressed as the highest dilution of plasma or immunoglobulins which resulted in a 50% reduction of luciferase luminescence compared with virus control wells.

### RNA Extraction and RNA Sequencing

The total RNA of whole blood from 1 COVID-19 patient with SARS-CoV-2 RNA retested positivity, nine mild-to-moderate (MM) patients, two severe patients, and four healthy donors were extracted using Trizol reagents (Invitrogen, USA). The sequencing libraries were prepared according to instructions of the Illumina TruSeq RNA Sample Preparation kit (Illumina, USA). Sequencing was performed on a NovaSeq 6000 system (Illumina, USA). These experiments were performed by DianLi Bio-Tec Co., Ltd (Guangzhou, China). The mRNA sequencing datasets have been deposited in the Genome Sequence Archive for Human (https://bigd.big.ac.cn/gsa-human/) with the accession number HRA000661.

### Pre-Processing of Raw RNA-Seq data

Raw RNA-seq reads were filtered according to their base qualities, and read sequences were trimmed at the 3’-end after reaching a 2-base sliding window with a PHRED quality score lower than 20. Following filtering, Illumina adapter sequences at the 3’-end were removed using Trimmomatic v0.36 (18). After low-quality filtering and adapter trimming, reads of more than 50 nt in length were retained for further analysis. Next, the trimmed reads were mapped to the human genome reference hg38 using HISAT v2.1 (19) with corresponding gene annotations (Gencode GRCh37/V32 for the human genome) at default settings. Total counts per mapped gene were determined using featureCounts function in SubReads package v1.5.3 (20) with default parameter.

### Global Transcriptome Analysis and Digital Cytometry

To determine the difference of whole blood transcriptome between COVID-19 patients with different severity, normalized counts matrix was used to calculate pairwise person coefficients using the build-in function cor in R software. Hierarchical clustering across all samples was based on pairwise Pearson correlation coefficients matrix. The build-in function prcomp in R software was used for principal component analysis (RCA). We inferred the immune cell quantities in each blood sample using the CIBERSORT server (https://cibersortx.stanford.edu/).

### Identification of Differentially Expressed Genes and Functional Enrichment Analysis

Raw counts matrix obtained from featureCounts were used as input for differentially expressed gene analysis using the Bioconductor package DESeq2 v1.26 (21) in R v3.6. Gene counts of more than five reads in a single sample or more than 100 total reads across all samples were retained for further analysis. Filtered counts matrix was normalized using the DESeq2 method to remove the library-specific artefacts. Genes with log2 fold change >1 or <−1 and adjusted *p*-value <0.05 corrected for multiple testing using the Benjamini–Hochberg (BH) method were considered significant. Gene ontology analysis was performed to assess their biological relevance using R package clusterprofiler v3.14.3 (22).

### Statistical Analysis

Statistical analyses and graphical presentations were conducted using GraphPad Prism v5.0 (GraphPad Software, Inc., CA, USA). Correlation analyses were performed using the Spearman correlation test. Differences of immune cell counts between the re-tested positivity (RP) patient, non-RP MM patients, and severe patients, NAbs titer between anti-spike/anti-RBD-antibody IgA or IgG positive and negative group were determined using the Wilcoxon signed-rank test. *p <*0.05 was considered statistically significant.

## Results

### The Clinical Characteristics of COVID-19 Patients

**Figure 1:**
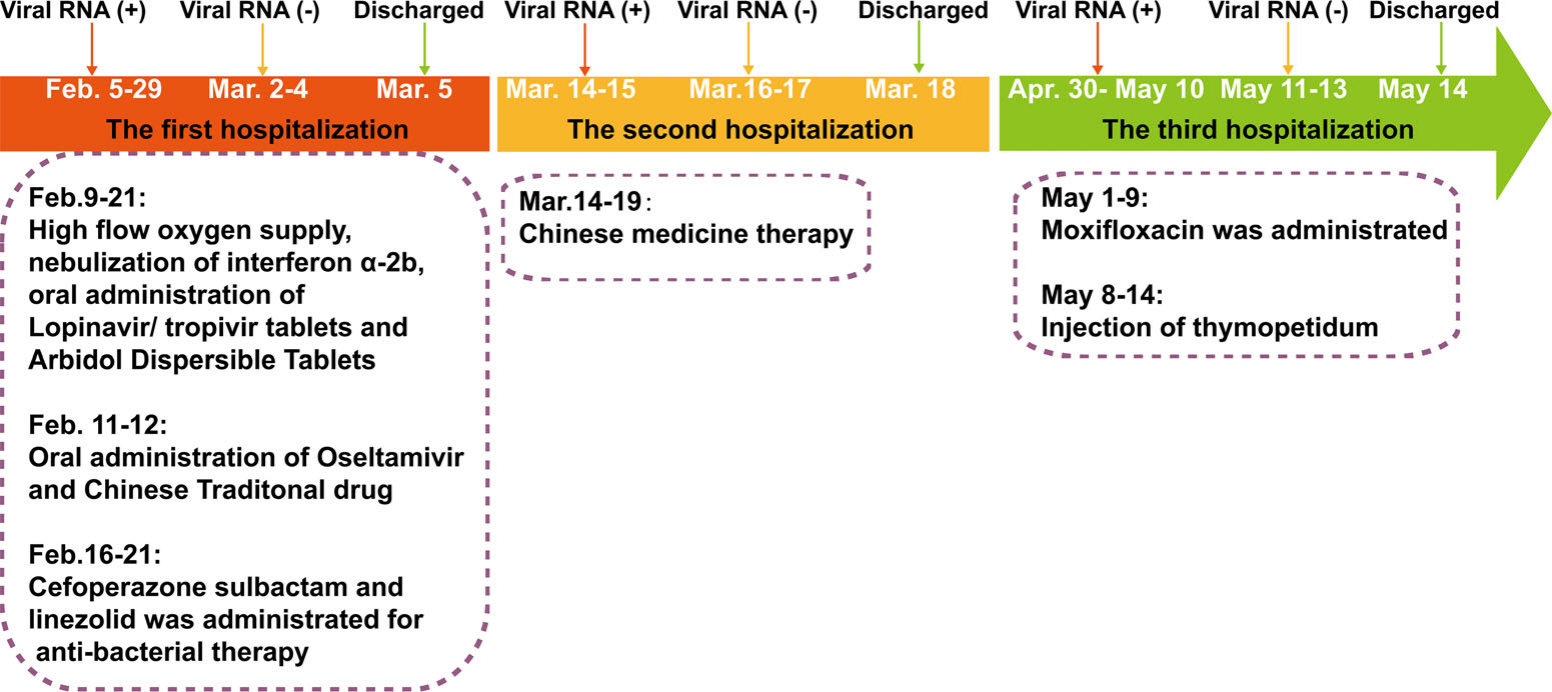
The severe acute respiratory syndrome coronavirus-2 (SARS-CoV-2) reverse transcriptase-PCR test results and clinical therapy of the SARS-CoV-2 RNA re-tested positivity patient during hospitalization period.

From January 27, 2020, to March 5, 2020, there were 15 patients with confirmed COVID-19 in Maoming city (**Table 1**). By March 5, 2020, all the patients were discharged and continued self-isolation based on the discharge criteria of China’s COVID-19 epidemic prevention and control strategy (15). Among them, one patient was re-tested positive for SARS-CoV-2 RNA and was re-admitted to the hospital twice during post-discharge surveillance (from March 5, 2020, to July 1, 2020) (**Table 1** and **Figure 1**). However, all the close contact family members of the re-admitted patient were negative for SARS-CoV-2 RNA and antibody following long surveillance (from January 21, 2020, to July 1, 2020), implying the re-tested SARS-CoV-2 RNA positivity of this patient was not caused by re-infection from his close contacts, and representative of SARS-CoV-2 RNA prolonged positivity.

**Table 1:** The clinical characteristics of the COVID-19 patients in Maoming City.

No.	Gender	Age	Clinical Classification	The time from Wuhan to Maoming	Diagnosis date	Discharge date	RNA positive duration (days)	Comorbidities	Symptoms	Pneumonia	PaO_2_/FiO_2_ (mmHg)
S1	Male	53	Severe	Jan.23,2020*	Feb.2,2020	Mar.1,2020	25	Hypertension	Chills and fever	Yes	235
S2	Male	53	Severe	Jan.21,2020	Feb.2,2020	Feb.18,2020	14	Hypertension	Fever, chills, cough	Yes	295
S3	Male	41	Severe	Jan.22,2020	Jan.27,2020	Feb.12,2020	13	No	Fever, stuffy nose and runny nose, muscle soreness, chills,	Yes	265
RP	Male	60	Moderate	Jan.22,2020	Feb.5,2020	Mar.5,2020	95	CAE, Hypertension	Fever	Yes	318
M1	Male	23	Moderate	Jan.21,2020	Feb.3,2020	Feb.27,2020	21	No	Cough, Weakness	Yes	476
M2	Male	40	Moderate	Jan.22,2020	Jan.26,2020	Mar.5,2020	37	No	Fever, Cough, Runny nose, Stuffy nose	Yes	391
M3	Female	68	Moderate	Jan.21,2020	Feb.7,2020	Mar.5,2020	25	Hypertension, DM	No	Yes	326
M4	Female	18	Moderate	Jan.20,2020	Feb.4,2020	Feb.18,2020	12	No	No	Yes	450
M5	Female	33	Moderate	Jan.19,2020	Feb.6,2020	Feb.18,2020	11	No	No	Yes	515
M6	Male	38	Moderate	Jan.21,2020	Feb.6,2020	Feb.27,2020	20	No	No	Yes	417
M7	Male	22	Moderate	Jan.22,2020	Feb.3,2020	Feb.14,2020	9	No	No	Yes	396
M8	Male	34	Mild	Jan.22,2020	Feb.13,2020	Mar.3,2020	16	No	Weakness	No	460
M9	Male	26	Mild	Jan.21,2020	Feb.3,2020	Feb.24,2020	30	No	Fever	No	396
M10	Male	16	Mild	Jan.22,2020	Jan.29,2020	Feb.7,2020	8	No	Fever	No	620
AI	Male	22	AI	Jan.22,2020	Jan.26,2020	Feb.7,2020	8	No	No	No	No data

*infected by YXY(S2); ^#^infected by his sun LWX(M6); S, severe; M, mild-to-moderate; AI, asymptomatic infection; CAE, Coronary artery embolism; DM, Diabetes mellitus.

The RP patient had a history of hypertension and coronary artery interventional therapy and was diagnosed with COVID-19 on February 5, 2020 (**Figure 1**). On admission, the RP patient had no apparent symptoms and was diagnosed as moderate COVID-19 infection. He was treated with daily doses of anti-interferon α-2b, lopinavir/tropivir tablets, arbidol dispersible tablets and high flow oxygen supply from February 9, 2020, to February 21, 2020. On February 16, 2020, he had a transient high fever (38.8°C) and was treated with cefoperazone sulbactam and linezolid until the lesions in the right lower lung, which were first observed on February 8, 2020, was absorbed (February 21, 2020). The RP patient was discharged on March 5, 2020, based on discharged criteria guidelines (15). However, the patient was re-tested positive for SARS-CoV-2 RNA and was re-admitted to the hospital on March 14, 2020 (**Table 1** and **Figure 1**). After treatment using Chinese traditional medicines, he was then discharged (March 18, 2020) based on continuous negative SARS-CoV-2 RNA results for 3 days. Appallingly, SARS-CoV-2 RNA was redetectable from oropharyngeal swab sample on April 30, 2020. Collectively, the intermittent viral RNA positivity of this patient lasted from February 5, 2020, to May 10, 2020 (~95 days), and was significantly longer than that of previously reported cases (23). The plasma IgA, IgG and IgM concentration of the RP patient was consistently normal during the SARS-CoV-2 RNA positive and negative stage (**Supplemental Table 1**). Analysis of immune cell types and their proportions based on transcriptome data indicated that CD4^+^ and CD8^+^ T cells, and B cells of the patient at the recovery stage were similar with healthy controls (24) (**Figure S1**). In addition, the RP patient was tested negative for HIV, HBV, HCV, syphilis, tuberculosis, and cancer; indicating that the patient was immunocompetent.

**Figure 2:**
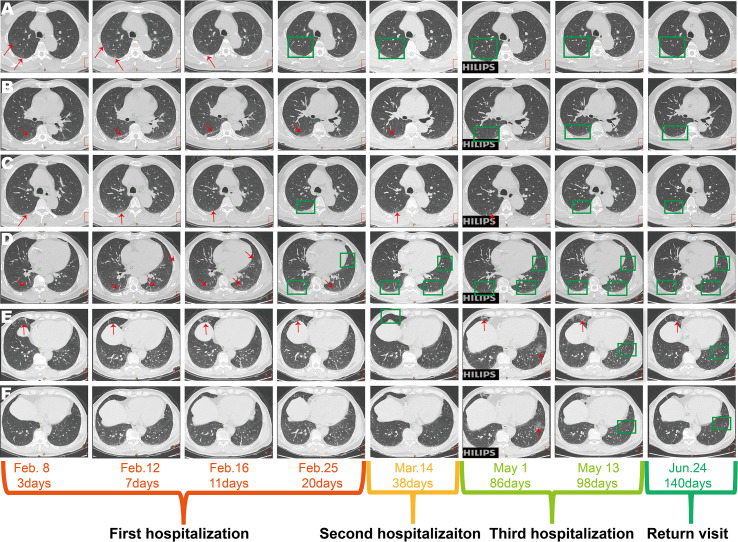
Chest computed tomography scans of the COVID-19 patient with SARS-CoV-2 RNA re-tested positivity during the hospitalization period. **(A)** The ground-glass opacities in the upper part of the posterior segments of the right upper lobe. **(B)** The ground-glass opacity in the posterior segments of the right lower lobe. **(C)** The ground-glass opacity in the lower part of posterior segments of the right upper lobe disappeared in the first hospitalization, re-appeared in the second and third hospitalization (the first recurrent SARS-CoV-2 RNA positive and the second recurrent SARS-CoV-2 RNA positive). **(D)** The ground-glass opacity in the right upper lobe, left lower lobe, and lingual segment of the left upper lobe. **(E)** The lesion of the right middle lung displayed as patchy, ground-glass and linear lesion during the first hospitalization, disappeared during the second hospitalization, re-appeared and was enlarged during the third hospitalization, but was absorbed on the return visit (June, 24, 2020). **(F)** Appearance of the ground-glass opacity in the basal segment of the left lower lung near the diaphragmatic dome at third hospitalization (the second recurrent SARS-CoV-2 RNA positive).

Longitudinal chest computed tomography (CT) demonstrated ground-glass opacities in the posterior segments of the right upper lobe, posterior segments of the right lower lobe, left lower lobe, and the lingual segment of the left upper lobe were observed in the early stage of the first hospitalization (from February 8, 2020, to February 16, 2020), disappeared gradually at the first discharge (February 25, 2020) (**Figures 2A–D**). However, the ground-glass opacities in the lower part of the posterior segments of the right upper lobe reappeared in the second (first SARS-CoV-2 RNA RP, March 14, 2020) and third admission (second SARS-CoV-2 RNA RP, May 1, 2020) and disappeared by the last discharge date (May 13, 2020) (**Figure 2C**). The patchy, linear, and ground-glass lesion in the right middle lobe was present from February 8, 2020, to February 25, 2020, disappeared in the second hospitalization (March 14, 2020) but re-appeared and was larger during the third hospitalization (from May 1, 2020, to May 13, 2020), then decreased by the return visit (June 24, 2020) (**Figure 2E**). Interestingly, new lesions in the basal segment of his left lower lobe emerged on the second admission (May 1, 2020) but vanished by the last discharge date (May 13, 2020) (**Figures 2E, F**).

### Delayed NAbs Conversion and Anti-Nucleocapsid (NP) IgM Persistent Positive Are Related to Prolonged SARS-CoV-2 RNA Positive

**Figure 3:**
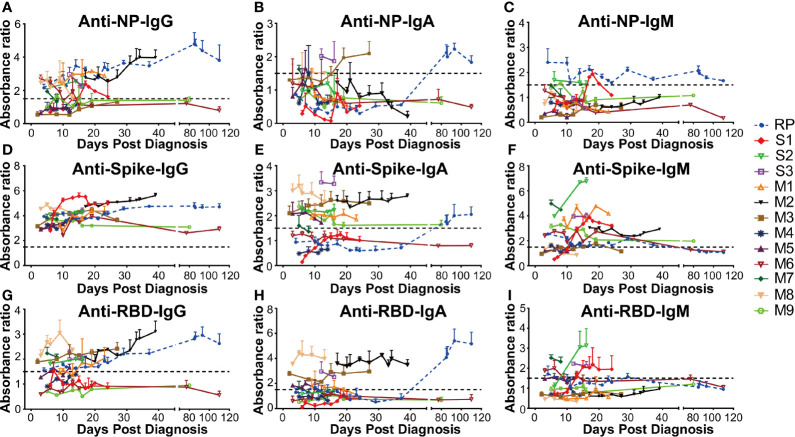
The dynamic change of anti-SARS-CoV-2 antibody in all COVID-19 patients. The absorbance ratio was calculated by the absorbance value of COVID-19 patients divided by 2.1 × mean absorbance value of four SARS-CoV-2 RNA negative plasma pool samples. **(A)** Anti-nucleocapsid (NP) IgG; **(B)** Anti-NP IgA; **(C)** Anti-NP IgM; **(D)** Anti-spike IgG; **(E)** Anti-spike IgA; **(F)** Anti-spike IgM; **(G)** Anti-receptor-binding domain (RBD) IgG; **(H)** Anti-RBD IgA; **(I)** Anti-RBD IgM. RP, re-tested positivity; M, Mild-to-moderate; S, Severe.

The antibody levels in 13 SARS-CoV-2 RNA positive patients including one RP patient, nine MM patients (M1–M9), and three severe patients (S1–S3) were comparatively explored. Our data demonstrated that the anti-NP antibody IgG in three MM patients (M3, M4, and M9) were persistently negative. The M6 patient had early seroconversion of anti-NP antibody IgG but turned negative ten days later, while for the remaining patients, anti-NP antibody IgG was persistently positive (**Figure 3A**). M3, M8, and S3 patients had early seroconversion of anti-NP antibody IgA. The RP patient had delayed seroconversion of anti-NP antibody IgA while the remaining patients were persistently negative (**Figure 3B**). Three patients (S1, S2, and RP) had seroconversion of anti-NP antibody IgM, S1, and S2 patients, which turned negative in 7 and 10 days. The RP patient had persistently detectable anti-NP antibody IgM for 110 days (February 9, 2020, to May 25, 2020), while the remaining patients remained persistently negative (**Figure 3C**). Given the key role of NP in the SARS-CoV-2 genome replication (25), the long duration for anti-NP IgM positivity could suggest persistent proliferation of SARS-CoV-2 in the RP patient.

In addition, all the patients were persistently positive for anti-spike antibody IgG during their first hospitalization (**Figure 3D**). However, the anti-spike antibody IgA level varied between different patients, of whom two MM patients (M4 and M6) were persistently negative, other MM patients (M1, M2, M3, M5, M7, M8) had persistent positivity, two severe patients (S2 and S3) were persistently positive, and one severe patient (S1) was persistently negative during hospitalization. The RP patient had delayed seroconversion of anti-spike antibody IgA, compared to the other IgA positive patients (**Figure 3E**). As for anti-RBD antibody IgA and IgG, six MM(M1, M2, M3, M5, M7, M8) and one severe patient (S3) had early seroconversion (**Figures 3G, H**). Three MM (M4, M6, M9) patients and one severe patient (S1) were persistently negative for anti-RBD antibody IgA and IgG. One severe (S2) patient displayed early seroconversion of anti-RBD antibody IgG but was persistently negative for anti-RBD antibody IgA. The RP patient was positive for anti-RBD antibody IgG during the early infection stage but then had delayed seroconversion of anti-RBD antibody IgA (**Figures 3G, H**). Additionally, the majority of the COVID-19 patients had early seroconversion of anti-spike IgM antibody, with three MM (M3, M5, M8) patients being marginally positive (**Figure 3F**). Among them, three severe patients (S1, S2, and S3) and two MM (M6, M7) patients were positive for anti-RBD IgM antibodies. The RP patient was positive at the early infection stage and turned negative at the convalescent stage, while the others were negative for anti-RBD IgM antibody (**Figure 3I**).

**Figure 4:**
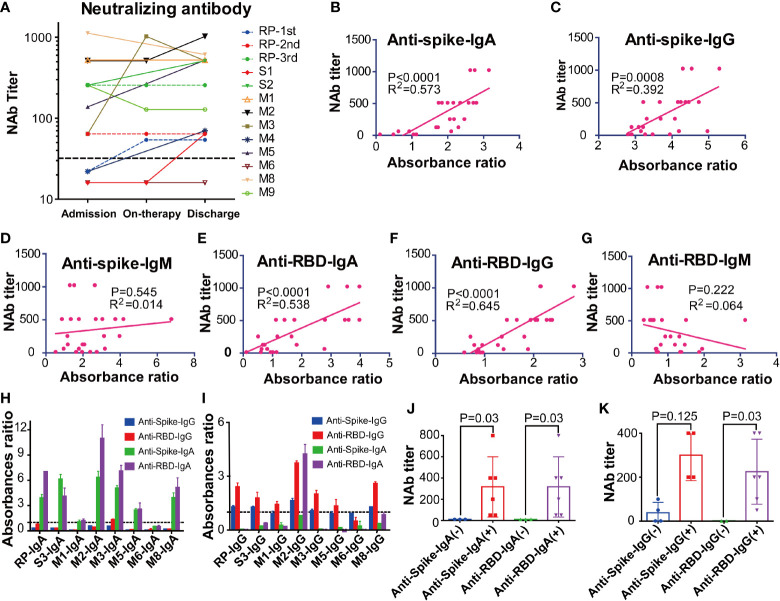
The kinetic and correlation of anti-SARS-CoV-2 antibody and neutralizing antibody (NAbs) of all COVID-19 patients. **(A)** The dynamic change of the neutralizing antibody (NAbs) of all COVID-19 patients. **(B–G)** The correlation between NAbs level and anti-spike IgA(B), anti-spike IgG **(C)**, anti-spike IgM **(D)**, anti-receptor-binding domain (RBD) IgA **(E)**, anti-RBD IgG **(F)**, anti-RBD IgM **(G)** in all COVID-19 patients. The cut-off for the pseudovirus neutralization assay is 1:50. **(H–I)** The absorbance ratios of anti-spike an anti-receptor binding domain (RBD) specific antibody in the isolated total IgA **(H)** and IgG **(I)**, that were calculated by the absorbance value of antibody isolated samples divided by 2.1 × mean absorbance value of anti-SARS antibody-negative samples (M6). **(J)** The neutralization antibody (NAbs) titers of the anti-spike/anti-RBD antibody IgA positive and negative groups. **(K)** The neutralization antibody (NAbs) titers of anti-spike/anti-RBD antibody IgG positive and negative groups. RP, re-tested positivity; MM, Mild-to-moderate; S, Severe.

The NAbs level of eleven patients on admission and discharge was further evaluated. Our results showed that the NAbs level varied between different patients. Particularly, the RP patient had low NAbs levels at the first and second discharge but had high NAbs levels at the third admission (**Figure 4A**). Interestingly, the high NAbs levels in the RP patient was accompanied with a sharp increase of anti-RBD/anti-Spike IgA and gradual rise of anti-RBD/anti-spike IgG (**Figures S2A–C**). The high NAbs levels of the RP patient lasted at least 78 days (from April 30, 2020, to July 17, 2020) (**Figure S2C**) and the NAbs levels in the RP patient was positively associated with anti-spike/anti-RBD IgA and IgG, and was negatively associated with anti-spike/anti-RBD IgM (**Figures S2D–I**). Correlation analysis of all COVID-19 patients further suggested that their plasma NAbs level was positively associated with anti-RBD or anti-spike IgG and IgA, but not with anti-spike/anti-RBD IgM (**Figures 4B–G**). Next, total IgA and IgG were extracted from the anti-RBD antibody IgA positive plasma (**Figures 4H, I**). Neutralization assay demonstrated that anti-RBD/anti-spike IgA and anti-RBD IgG antibodies had SARS-CoV-2 virus neutralizing capacity but the anti-spike IgG antibody positivity could not represent SARS-CoV-2 virus NAbs levels (**Figures 4J, K**).

### Impaired T Cells and Delayed B Cells Response Are Related to Prolonged SARS-CoV-2 RNA Positivity

**Figure 5:**
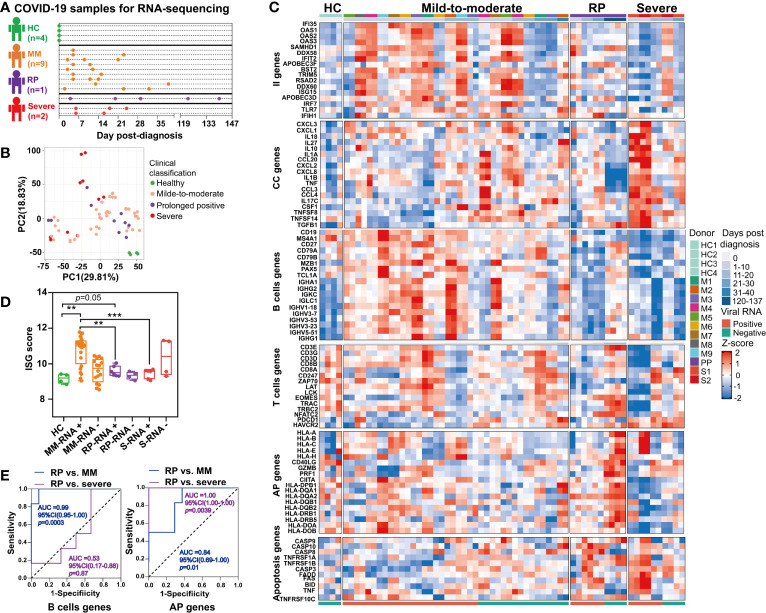
Comparison of immune signatures between the SARS-CoV-2 RNA re-tested positivity (RP) patient and Mild-to-moderate (MM) patients or severe patients. **(A)** Schema of the RNA-seq experimental design. A total of 12 COVID-19 patients and four healthy controls (HC) were included in this study. **(B)** Unsupervised principal component analysis based on global gene expression. **(C)** Heatmap of a subset of innate immunity (II) genes, cytokine and chemokine (CC)-related genes, B cells-related genes, T cells-related genes, antigen presentation (AP) genes (including MHC class I-related genes and MHC class II-related genes) and apoptosis-related genes. **(D)** Comparison of interferon-stimulated genes (ISGs) scores between the PP patient and MM patients or severe patients. Asterisks represent significant differences between groups (**p-value < 0.01, ***p-value < 0.001, unpaired t-test). **(E)** Receiver-operating characteristic curve of the clinical course prediction based on the expression of B cells-related genes and antigen presentation (AP)-related genes.

To investigate the immunological mechanisms underlying prolonged SARS-CoV-2 persistence, we further compared the immune signatures of one RP patient, nine MM patients, two severe patients and four healthy donors *via* RNA-seq of their whole blood (**Figure 5A**).

Unsupervised PCA revealed that the gene expression profile of whole blood in healthy controls formed a distinct cluster whereas that in COVID-19 patients formed clusters that were more widely dispersed and overlapped, indicating the immune heterogeneity in different COVID-19 patients (**Figure 5B**). Further, our results demonstrated that the RP patient and severe patients had more markedly declined expression of interferon-stimulated genes (ISGs) in the acute infection stage (**Figure 5C**). Additionally, the ISG score, i.e. the mean expression of six ISGs (*IFI27*, *IFI44L*, *IFIT1*, *RSAD2*, *SIGLEC1*, and *ISG15*) defining a type I IFN response was significantly lower in the RP patient and severe patients compared to MM patients (**Figure 5D**) (26). Consistent with previous report (27), we also observed overexpression of cytokines and chemokines (CC)-related genes in the severe COVID-19 patients during their early stage of infection, including pro-inflammatory cytokines or chemokines such as *IL1A*, *IL1B*, *IL10*, *TNF*, *CCL20*, *CCL3*, *CCL4*, *CXCL1*, *CXCL2*, *CXCL3*, and *CXCL8* (**Figure 5C**). In contrast to severe COVID-19 patients, the MM patients or RP patient exhibited a lower expression of CC-related genes (**Figure 5C**).

Interestingly, a delayed B cells response was found in the RP patient, contrasting to a robust rapidly induced B cells response in MM patients. As for severe patients, the B cells-related genes were at a consistently low expression (**Figure 5C**). Notably, the expression of several immunoglobulin heavy chain variable region genes such as *IGHV3-23*, *IGHV3-7*, *IGHV1-18*, *IGHV3-53*, and *IGHV5-51* were significantly lower in the RP patient and severe patients than in MM patients (**Figure 5C**). Particularly, *IGHV3-53*, the most frequently used IGHV gene for targeting the receptor-binding domain (RBD) of the spike protein (28), was lowly expressed in the RP patient. T cells-related genes in the RP patient were lowly expressed, compared to healthy controls, until SARS-CoV-2-negative time points. Unlike the RP patient, lower expression of the T cells-related genes only lasted for 10–12 days in the severe patients, whereas a continuous relative normal T cells response was observed in most MM patients. In addition, T cells exhaustion was observed in severe COVID-19 patients but not in the RP or MM COVID-19 patient, as supported by the distinct expression pattern of T cells exhaustion-related genes such as programmed cell death 1 (*PD-1*, *PDCD1*) and Tim-3 (*HAVCR2*) (**Figure 5C**).

Compared to healthy controls, we observed an up-regulation in both MHC class I and MHC class II gene in the RP patient during the SARS-CoV-2-negative period, whereas this observation was not found during the SARS-CoV-2-positive period. In contrast to the RP patient, severe patients had decreased expression of MHC class II genes (**Figure 5C**). Notably, we observed an increased expression of *GZMB* and *PRF1* in the RP patient during his convalescent stage (**Figure 5C** **)** which acted as cytotoxic effectors of CD8^+^ T cells, CD4^+^ T cells, and NK cell (29). In addition, genes related to *Fas*- or *TNF*-linked apoptosis pathways showed a higher expression in the RP and severe patients compared to MM patients during the SARS-CoV-2-positive period (**Figure 5C**). Moreover, the B cells proportion at the SARS-CoV-2-positive stage in the RP patient was significantly lower than healthy controls, MM and severe patients (**Figure S1B**). The CD4^+^ T cells subsets proportions were increased in all three clinical-classification patients at viral RNA positive stage, and returned to normal in the RP patient during viral RNA negative stage (**Figure S1C**). The CD8^+^ T cells subsets showed different degrees of decline in all three clinical-classification patients at viral RNA positive stage, and returned to normal in the RP patient during viral RNA negative stage (**Figure S1D**).

To determine whether these gene expression patterns could predict clinical course, we performed a prediction analysis based on the expression of the six gene modules. We found that the expression of B cells-related genes could distinguish the RP patient from MM patients, but not from the severe patients (**Figure 5E**). Interestingly, the expression of MHC-related genes could distinguish the RP patient from MM or severe patients, i.e. the downregulation of MHC-related genes was related to severe COVID-19, while the upregulation of MHC-related genes was associated with MM COVID-19 and unaltered expression of MHC-related genes was linked to prolonged SARS-CoV-2 RNA shedding (**Figure 5E**).

### Longitudinal Transcriptome Analysis Revealed the Dynamic Immune Response in the Patient With Prolonged SARS-CoV-2 RNA Positive

**Figure 6:**
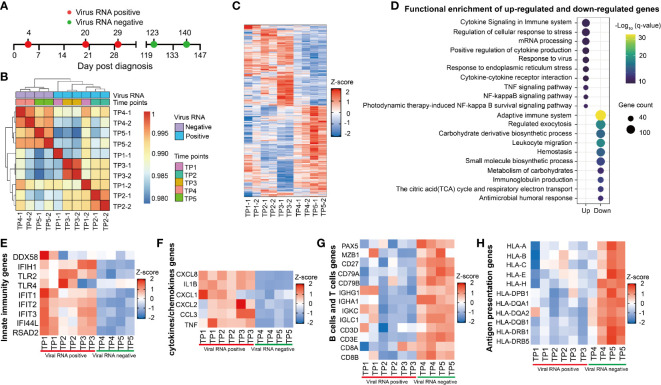
Longitudinal transcriptional profile of whole blood in the SARS-CoV-2 RNA re-tested positivity (RP) patient. **(A)** Samples of whole blood collected at five different time points were used for RNA sequencing. **(B)** Heatmap of the correlation coefficient matrix across different time points. **(C)** Heatmap of the unique different expression genes (DEG) identified over time. **(D)** Functional enrichment analysis of up-regulated (top) or down-regulated gene (bottom) at TP1-TP3 comparing to TP4 and TP5. Color denotes statistical significance as measured by minus logarithm of p-values. **(E–H)** Heatmap of DEGs related to innate immunity response **(E)**, cytokines or chemokines **(F)**, B cells and T cells **(G)**, and antigen presentation **(H)** during viral positive and negative period.

We then investigated the kinetic immune response in the RP patient by RNA sequencing (RNA-seq) of his whole blood samples covering five-time points (TP1-TP5) (**Figure 6A**). Correlation analyses showed a distinct global gene expression profile between SARS-CoV-2-positive (TP1-TP3) and SARS-CoV-2-negative (TP4-TP5) samples (**Figure 6B**). Compared to TP4 and TP5, we identified 1,418 different expression genes (DEGs) at TP1, 2461 DEGs at TP2, 4,092 DEGs at TP3 respectively, indicating a dramatically disturbed transcriptome homeostasis of peripheral blood cells at SARS-CoV-2 RNA positive stage (**Figure 6C**). Functional enrichment analysis showed that the up-regulated genes were mainly associated with cytokine related signaling pathway and mRNA processing whereas the down-regulated genes were related to adaptive immune and humoral immune response (**Figure 6D**).

The innate immunity genes involving in virus detection (*DDX58*, *IFIH1*, *TLR2*, and *TLR4*) and induction of antiviral effectors (*IFIT1*, *IFIT2*, *IFIT3*, *IFI44L*, and *RSAD2*) were upregulated in the RP patient during viral RNA positive stage (**Figure 6E**). This result indicated that *IFN*-mediated broad-spectrum antiviral functions were induced in the RP patient. In addition, an increased production of pro-inflammatory mediators such as *CXCL8*, *IL1B*, *CXCL1*, *CXCL2*, *CCL3*, and *TNF* were observed during the viral RNA positive stage (**Figure 6F**), suggesting an unquenched inflammation response during viral persistence in the RP patient.

Compared to SARS-CoV-2-negative time points (TP4 and TP5), we observed a lower expression of B cells-related genes, including B cells transcription and activation regulators related genes (*PAX5*, *MZB1*, *CD27*, *CD79A*, and *CD79B*), and immunoglobulin producible genes (*IGHG1*, *IGHA1*, *IGKC1*, and *IGLC1*) in SARS-CoV-2-positive time points (TP1-TP3) (**Figure 6G**). In addition, the expression of T cells-related genes (*CD4*, *CD3E*, *CD8A*, and *CD8B*) were lower in the viral RNA positive stage (**Figure 6G**). Furthermore, the expression of MHC class I genes (*HLA-A*, *HLA-B*, *HLA-C*, *HLA-E*, and *HLA-H*) and MHC class II genes (*HLA-DPB1*, *HLA-DQA1*, *HLA-DAQ2*, *HLA-DQB1*, *HLA-DRB1*, and *HLA-DRB5*) were lower in SARS-CoV-2-positive samples (TP1–TP3) (**Figure 6H**). Further, we found that the expression of MHC, B cells and T cells-related genes was negatively correlated with viral RNA concentration and the CC-related genes were positively associated with viral RNA concentration (**Figure S3**).

## Discussion

In general, it is reported that SARS-CoV-2 RNA shedding last for 18–25 days in immunocompetent patients (30). In this present study, the immunocompetent RP patient experienced ~95 days of prolonged virus RNA shedding; longer than the longest previously reported 56 days period (23). The RP patient had a new lesion in his lung during the re-detectable RNA positive stage (the second and third admissions) but all the close contact family members of the patient were not infected and were tested negative for SARS-CoV-2 RNA and antibody following a long surveillance; suggesting the previous infected SARS-CoV-2 isolate was not completely cleared during the first discharge. Nevertheless, the RP patient could not be excluded for re-infection of novel mutated SARS-CoV-2 isolates because prolonged infection of SARS-CoV-2 could accelerate its mutation in immunocompromised hosts (5). In contrast to the RP patient in this study, previous study showed that 44 SARS-CoV-2 RNA re-positive cases had no ground glass opacities during viral RNA re-positive period (15), implying the immunity mechanisam underlying viral RNA re-tested positivity in RP patient with novel lung lessions in our study might not be generalized to other RP patients without novel lung lessions.

Although, an upregulation of anti-spike antibody IgA, anti-RBD antibody IgA and NAbs was followed by the complete clearance of SARS-CoV-2 RNA and the lung lesion in the immunocompetent patient during the last hospitolization, the times of seroconversion of anti-RBD IgA and NAbs were indefinite because of a long sampling void between the second (March 5, 2020) and third hospitalization (May 1, 2020). If seroconversion of NAbs occurred during the sampling viod period, suggesting NAbs was not crucial in the clearance of the lung lesions. However, previous data (31) and our experimental data confirmed that anti-RBD antibody IgG and the anti-RBD/anti-spike antibody IgA content was correlated with virus-neutralizing capacity. A recent study demonstrated an inverse correlation between the nasopharyngeal swabs viral load and the plasma level of anti-S1 IgA in a severe patient (32). Moreover, the plasma titer of anti-S1 IgA was a protective factor of day-28 mortality of severe patients (32). Together, the anti-RBD antibody IgA and IgG level are important immunity response evaluation biomarker for SARS-CoV-2 vaccine development (33).

Of note, the delayed plasma anti-SARS-CoV-2 antibody IgA conversion and NAbs production occurred in the RP patient. Moreover, lower levels and slower generation of anti-RBD-specific IgA and IgG were reported to contribute to SARS-CoV-2 RNA persistence in the gastrointestinal tract (34). This unique phenomenon may be related to lagged activation of the MHC class II-mediated antigen presentation-related genes upon SARS-CoV-2 infection in the RP patient. In addition, the unchanged expression of antigen presentation-related genes in the early stage of infection could be a prediction of delay SARS-CoV-2 clearance. On the other hand, the RP patient had lower B cells during his acute infection stage than non-RP MM patients. All these could have contributed to the observed delayed humoral immune response.

The IFN response occurred in the RP patient was lower than in the MM COVID-19 patients and higher than in severe COVID-19 patients, suggesting that limited IFN response was insufficient to timely clear the virus. Moreover, the CC-related gene expression of the RP patient was lower than severe patients, not different from non-RP MM patients. The low CC-related genes explain why the RP patient did not progress to severe disease (35).

Herein, evidences indicated severe patients had the lowest T cells gene expression and recovered in a short time. Accordingly, exhaustion-related genes were most upregulated in severe patients as compared to healthy controls, implying that the dysregulated function of T cells could be related to disease severity (36). However, a sustained low expression of T cells-related gene and overexpression of exhaustion-related genes in the SARS-CoV-2 positive stage of the RP patient, indicating that impaired T cell responses also contributed to viral persistence. Theoretically, the impaired T cell response may be attributed to the downregulated MHC class I genes, upregulated *Fas*- or *TNF*-linked apoptosis pathways, and decreased B cells and CD8^+^ T cells in the RP patient. In the convalescent stage, the RP patient demonstrated higher expression of T cells, MHC class I and II related genes, and upregulation of NAbs; suggesting coordinated T cells immunity and humoral immunity response played a crucial role in viral clearance of the RP patient at the convalescent stage (13).

In conclusion, our study showed that low innate immune response and prolonged low antigen presentation capacity were related to the re-tested SARS-CoV-2 RNA positivity in this immunocompetent patient. In the convalescent stage of this RP patient, upregulation of the antigen presentation gene might have contributed to the coordinated humoral and cellular immune response and led to SARS-CoV-2 clearance.

## Data Availability Statement

The datasets presented in this study can be found in online repositories. The names of the repository/repositories and accession number(s) can be found below: Genome Sequence Archive for Human, accession no: HRA000661.

## Ethics Statement

The studies involving human participants were reviewed and approved by the Ethics Committee of Maoming People’s Hospital (No. 2020012). The patients/participants provided their written informed consent to participate in this study. Written informed consent was obtained from the individual(s) for the publication of any potentially identifiable images or data included in this article.

## Author Contributions

Conceptualization: TJ. Clinical sample and data collection: CL, HL, LZ, YC, YFC, WH, FX, LHH, YL, JH, LL and, HZ. Methodology devising and Performing experiments and Data analysis: TJ, XL, QY, LBH, ZL, and YX. Writing original draft: TJ, QY, XL, GW, and QZ. Funding acquisition: TJ, CC, QZ, GW, and HL. Supervision: LC, ZZ, CC, and TJ. All authors contributed to the article and approved the submitted version.

## Funding

This study received the following funding: Guangdong Science and Technology Project (2014A020212331), Guangdong Provincial Department of Education Youth innovative talents Project (2017KQNCX168), Guangzhou Health Science and technology project (20201A011078), Guangzhou Science and Technology Project (201904010214, 202102010094), Emergent Science and Technology Project for Prevention and Treatment of COVID-19 disease (#mmkj003) and the High-level Hospital Construction Research Project of Maoming People’s Hospital (zx2020012), Maoming Science and Technology Project (2020007), Guangdong Basic and Applied Basic Research Foundation (2021A1515012550), Natural Science Foundation of Guangdong Province (2019A1515011681), and Guangzhou Institute of Respiratory Health Open Project (2020GIRHHMS01).

## Conflict of Interest

YX was employed by Guangdong South China Vaccine Co. Ltd.

The remaining authors declare that the research was conducted in the absence of any commercial or financial relationships that could be construed as a potential conflict of interest.

## Supplementary Material

Supplementary Figure 1 Comparison of immunity cells counts between the SARS-CoV-2 RNA re-tested positivity (RP) patient and mild-to-moderate (MM) patients or severe patients. **(A)** The proportion of nine major immune cell types in the peripheral blood was determined using CIBERSORT. **(B–D)** Comparison of B cells **(B)**, CD4^+^ T cells **(C)**, and CD8^+^ T cells **(D)** between the SARS-CoV-2 RNA RP patient and MM or severe patients. Asterisks represent significant differences between groups (**p*-value < 0.05, ***p*-value < 0.01, ****p*-value < 0.001, unpaired t-test). HC, Healthy controls.

Supplementary Figure 2 The kinetic and correlation of anti-SARS-CoV-2 antibody and neutralizing antibody (NAbs) in the SARS-CoV-2 RNA re-tested positivity (RP) patient. The absorbance ratio was calculated by the absorbance value of COVID-19 patients divided by 2.1 × mean absorbance value of 4 SARS-CoV-2 RNA negative plasma pool samples. **(A–C)** The dynamic change of Anti-spike antibody **(A)**. Anti-receptor binding domain (RBD) antibody **(B)**, NAbs titer **(C)** in SARS-CoV-2 RNA RP patient. **(E–I)** The correlation between NAbs titer and anti-spike IgA **(D)**, anti-RBD IgA **(E)**, anti-spike IgG **(F)**, anti-RBD IgG **(G)**, anti-spike IgM **(H)**, and anti-RBD IgM **(I)** in SARS-CoV-2 RNA RP patient.

Supplementary Figure 3 Correlation between the Cycle threshold (Ct) value of viral RNA of COVID-19 patient with redetectable SARS-CoV-2 RNA and the expression of innate immunity (II) -related genes **(A)**, cytokine and chemokine (CC)-related genes **(B)**, B cells-related genes **(C)**, T cells-related genes **(D)**, apoptosis-related genes **(E)**, and antigen presentation-related genes **(F)**. The genes expression level was dertermited by comparing TP1, TP2, TP3, and TP4 with TP5 separately.

## References

[1] Wu J Liu X Liu J Liao H Long S Zhou N. Coronavirus Disease 2019 Test Results After Clinical Recovery and Hospital Discharge Among Patients in China. JAMA Netw Open (2020) 3(5):e209759. 10.1001/jamanetworkopen.2020.9759 32442288 PMC7244988

[2] Xing Y Mo P Xiao Y Zhao O Zhang Y Wang F. Post-Discharge Surveillance and Positive Virus Detection in Two Medical Staff Recovered From Coronavirus Disease 2019 (COVID-19), China, January to February 2020. Euro Surveill (2020) 25(10):2000191. 10.2807/1560-7917.ES.2020.25.10.2000191 PMC7078824 32183934

[3] Liu WD Chang SY Wang JT Tsai MJ Hung CC Hsu CL. Prolonged Virus Shedding Even After Seroconversion in a Patient With COVID-19. J Infect (2020) 81(2):318–56. 10.1016/j.jinf.2020.03.063 PMC7151379 32283147

[4] Zhang M Zhang J Shi H Liu B Zeng F. Viral Shedding Prolongation in a Kidney Transplant Patient With COVID-19 Pneumonia. Am J Transplant (2020) 20(9):2626–7. 10.1111/ajt.15996 PMC7272856 32400931

[5] Choi B Choudhary MC Regan J Sparks JA Padera RF Qiu X. Persistence and Evolution of SARS-CoV-2 in an Immunocompromised Host. N Engl J Med (2020) 383(23):2291–3. 10.1056/NEJMc2031364 PMC7673303 33176080

[6] Avanzato VA Matson MJ Seifert SN Pryce R Williamson BN Anzick SL. Case Study: Prolonged Infectious SARS-CoV-2 Shedding From an Asymptomatic Immunocompromised Cancer Patient. Cell (2020) 183(7):1901–12.e9. 10.1016/j.cell.2020.10.049 PMC7640888 33248470

[7] Niess H Borner N Muenchhoff M Khatamzas E Stangl M Graf A. Liver Transplantation in a Patient After COVID-19 - Rapid Loss of Antibodies and Prolonged Viral Rna Shedding. Am J Transplant (2020) 21(4):1629–32. 10.1111/ajt.16349 PMC7675727 33047475

[8] Zhu L Gong N Liu B Lu X Chen D Chen S. Coronavirus Disease 2019 Pneumonia in Immunosuppressed Renal Transplant Recipients: A Summary of 10 Confirmed Cases in Wuhan, China. Eur Urol (2020) 77(6):748–54. 10.1016/j.eururo.2020.03.039 PMC7166037 32317180

[9] Wu Q Chu Q Zhang H Yang B He X Zhong Y. Clinical Outcomes of Coronavirus Disease 2019 (COVID-19) in Cancer Patients With Prior Exposure to Immune Checkpoint Inhibitors. Cancer Commun (Lond) (2020) 40(8):374–9. 10.1002/cac2.12077 PMC7405291 32666636

[10] Shah V Ko Ko T Zuckerman M Vidler J Sharif S Mehra V. Poor Outcome and Prolonged Persistence of SARS-CoV-2 RNA in COVID-19 Patients With Haematological Malignancies; King’s College Hospital Experience. Br J Haematol (2020) 190(5):e279–82. 10.1111/bjh.16935 PMC7307054 32526039

[11] Karatas A Inkaya AC Demiroglu H Aksu S Haziyev T Cinar OE. Prolonged Viral Shedding in a Lymphoma Patient With COVID-19 Infection Receiving Convalescent Plasma. Transfus Apher Sci (2020) 59(5):102871. 10.1016/j.transci.2020.102871 32694044 PMC7333597

[12] Dong Z Yi H. An Integrated Genetic-Epigenetic Analysis Shed Light on the Mechanisms Linking Coronavirus Disease 2019 (COVID-19) and Cancer. Cancer Commun (Lond) (2021) 41(4):349–53. 10.1002/cac2.12151 PMC8014256 33636047

[13] Rydyznski Moderbacher C Ramirez SI Dan JM Grifoni A Hastie KM Weiskopf D. Antigen-Specific Adaptive Immunity to SARS-CoV-2 in Acute Covid-19 and Associations With Age and Disease Severity. Cell (2020) 183(4):996–1012 e19. 10.1016/j.cell.2020.09.038 33010815 PMC7494270

[14] Lin A He ZB Zhang S Zhang JG Zhang X Yan WH. Early Risk Factors for the Duration of Severe Acute Respiratory Syndrome Coronavirus 2 Viral Positivity in Patients With Coronavirus Disease 2019. Clin Infect Dis (2020) 71(16):2061–5. 10.1093/cid/ciaa490 PMC7197613 32337591

[15] Lu J Peng J Xiong Q Liu Z Lin H Tan X. Clinical, Immunological and Virological Characterization of COVID-19 Patients That Test Re-Positive for SARS-CoV-2 by RT-PCR. EBioMedicine (2020) 59:102960. 10.1016/j.ebiom.2020.102960 32853988 PMC7444471

[16] Guo L Ren L Yang S Xiao M Chang F Yang CS. : Profiling Early Humoral Response to Diagnose Novel Coronavirus Disease (Covid-19). Clin Infect Dis (2020) 71(15):778–85. 10.1093/cid/ciaa310 PMC7184472 32198501

[17] Feng L Wang Q Shan C Yang C Feng Y Wu J. An Adenovirus-Vectored COVID-19 Vaccine Confers Protection From SARS-COV-2 Challenge in Rhesus Macaques. Nat Commun (2020) 11(1):4207. 10.1038/s41467-020-18077-5 32826924 PMC7442803

[18] Bolger AM Lohse M Usadel B. Trimmomatic: A Flexible Trimmer for Illumina Sequence Data. Bioinformatics (2014) 30(15):2114–20. 10.1093/bioinformatics/btu170 PMC4103590 24695404

[19] Kim D Langmead B Salzberg SL. HISAT: A Fast Spliced Aligner With Low Memory Requirements. Nat Methods (2015) 12(4):357–60. 10.1038/nmeth.3317 PMC4655817 25751142

[20] Liao Y Smyth GK Shi W. featureCounts: An Efficient General Purpose Program for Assigning Sequence Reads to Genomic Features. Bioinformatics (2014) 30(7):923–30. 10.1093/bioinformatics/btt656 24227677

[21] Love MI Huber W Anders S. Moderated Estimation of Fold Change and Dispersion for RNA-seq Data With Deseq2. Genome Biol (2014) 15(12):550. 10.1186/s13059-014-0550-8 25516281 PMC4302049

[22] Yu G Wang LG Han Y He QY. clusterProfiler: An R Package for Comparing Biological Themes Among Gene Clusters. OMICS (2012) 16(5):284–7. 10.1089/omi.2011.0118 PMC3339379 22455463

[23] McKie AM Jones TPW Sykes C. Prolonged Viral Shedding in an Immunocompetent Patient With COVID-19. BMJ Case Rep (2020) 13(10). 10.1136/bcr-2020-237357 PMC7536772 33012717

[24] Newman AM Liu CL Green MR Gentles AJ Feng W Xu Y. Robust Enumeration of Cell Subsets From Tissue Expression Profiles. Nat Methods (2015) 12(5):453–7. 10.1038/nmeth.3337 PMC4739640 25822800

[25] Chowdhury UF Shohan MUS Hoque KI Beg MA Siam MKS Moni MA. A Computational Approach to Design Potential siRNA Molecules as a Prospective Tool for Silencing Nucleocapsid Phosphoprotein and Surface Glycoprotein Gene of SARS-Cov-2. Genomics (2020) 113(1 Pt 1):331–43. 10.1016/j.ygeno.2020.12.021 PMC7832576 33321203

[26] Hadjadj J Yatim N BarNAbsei L Corneau A Boussier J Smith N. Impaired Type I Interferon Activity and Inflammatory Responses in Severe COVID-19 Patients. Science (2020) 369(6504):718–24. 10.1126/science.abc6027 PMC7402632 32661059

[27] Arunachalam PS Wimmers F Mok CKP Perera R Scott M Hagan T. Systems Biological Assessment of Immunity to Mild Versus Severe COVID-19 Infection in Humans. Science (2020) 369(6508):1210–20. 10.1126/science.abc6261 PMC7665312 32788292

[28] Yuan M Liu H Wu NC Lee CD Zhu X Zhao F. Structural Basis of a Shared Antibody Response to SARS-Cov-2. Science (2020) 369(6507):1119–23. 10.1126/science.abd2321 PMC7402627 32661058

[29] Swain SL McKinstry KK Strutt TM. Expanding Roles for CD4(+) T Cells in Immunity to Viruses. Nat Rev Immunol (2012) 12(2):136–48. 10.1038/nri3152 PMC3764486 22266691

[30] Fontana L Villamagna AH Sikka MK McGregor JC. Understanding Viral Shedding of SARS-CoV-2: Review of Current Literature. Infect Control Hosp Epidemiol (2020) 42(6):659–68. 10.1017/ice.2020.1273 PMC7691645 33077007

[31] Marot S Malet I Leducq V Zafilaza K Sterlin D Planas D. Rapid Decline of Neutralizing Antibodies Against SARS-CoV-2 Among Infected Healthcare Workers. Nat Commun (2021) 12(1):844. 10.1038/s41467-021-21111-9 33558507 PMC7870823

[32] Fourati S Hue S Pawlotsky JM Mekontso-Dessap A de Prost N. Sars-CoV-2 Viral Loads and Serum IgA/IgG Immune Responses in Critically Ill COVID-19 Patients. Intensive Care Med (2020) 46(9):1781–3. 10.1007/s00134-020-06157-5 PMC7306494 32572527

[33] Hassan AO Kafai NM Dmitriev IP Fox JM Smith BK Harvey IB. A Single-Dose Intranasal ChAd Vaccine Protects Upper and Lower Respiratory Tracts Against SARS-Cov-2. Cell (2020) 183(1):169–184 e13. 10.1016/j.cell.2020.08.026 32931734 PMC7437481

[34] Hu F Chen F Ou Z Fan Q Tan X Wang Y. A Compromised Specific Humoral Immune Response Against the SARS-CoV-2 Receptor-Binding Domain is Related to Viral Persistence and Periodic Shedding in the Gastrointestinal Tract. Cell Mol Immunol (2020) 17(11):1119–25. 10.1038/s41423-020-00550-2 PMC7546387 33037400

[35] Varchetta S Mele D Oliviero B Mantovani S Ludovisi S Cerino A. Unique Immunological Profile in Patients With COVID-19. Cell Mol Immunol (2020) 18(3):604–12. 10.1038/s41423-020-00557-9 PMC7557230 33060840

[36] Zhou R To KK Wong YC Liu L Zhou B Li X. Acute SARS-CoV-2 Infection Impairs Dendritic Cell and T Cell Responses. Immunity (2020) 53(4):864–77.e5. 10.1016/j.immuni.2020.07.026 PMC7402670 32791036

