# Supplementary material for: Low Innate Immunity and Lagged Adaptive Immune Response in the Re-Tested Viral RNA Positivity of a COVID-19 Patient
Source: Front Immunol. 2021 Jul 1;12:664619. doi: 10.3389/fimmu.2021.664619 (PMC8295488; doi:10.3389/fimmu.2021.664619)
Supplement: Supplementary file 4 [file Table_1.doc]

**Supplemental table1. The plasma antibodies concentration of COVID-19 patient with SARS-CoV-2 RNA redetectable positivity**

| Antibody type | Feb.9,2020 | Feb.28,2020 | May 25, 2020 | Normal reference (g/L) |
| --- | --- | --- | --- | --- |
| IgA | 0.88 | 1.01 | 1.14 | 0.7-4.0 |
| IgG | 9.30 | 10.12 | 11.55 | 7.0-16.0 |
| IgM | 1.98 | 1.87 | 1.97 | 0.4-2.3 |
